# Supplementary material for: Are carbon emissions trading and green financial instruments synergistic? -Comprehensive quantitative research based on content analysis
Source: PLoS One. 2024 Mar 7;19(3):e0298601. doi: 10.1371/journal.pone.0298601 (PMC10919601; doi:10.1371/journal.pone.0298601)
Supplement: S1 Data — (ZIP) [file pone.0298601.s001.zip › Data/地级市Z_F工作报告.zip!/╜¡╦╒/┴1⁄4╘╞╕█╩╨/└┤╫╘╣1⁄2╓┌║┼í╛┬φ┐╦╩2╛▌═°í┐_2008.docx]

各位代表：

现在，我代表市人民政府向大会作工作报告，请予审议，并请市政协各位委员提出意见。

　过去五年工作回顾

过去的五年，是全市发展进程中不平凡的五年，是全市改革开放和全面小康社会建设取得巨大成就的五年。在中共连云港市委的领导下，本届政府以邓小平理论和三个代表重要思想为指导，认真落实科学发展观，紧紧依靠全市人民，解放思想，抢抓机遇，团结拼搏，全面完成十五计划，顺利实施十一五规划，实现了市十一届人民代表大会确定的各项奋斗目标。

一、加快产业结构调整，综合实力跨上新台阶

五年来，全市经济发展呈现总量扩张、增速加快、结构优化、质量提升的良好态势。2007年，预计实现地区生产总值615亿元，年均递增13.8%。人均生产总值13700元，是2002年的2.1倍。实现财政总收入122.8亿元，其中一般预算收入48.8亿元，均比2002年翻两番。累计完成全社会固定资产投资1796亿元，是前五年的2.8倍。三次产业结构由26.1:38.2:35.7调整为16.6:47:36.4。全面小康社会建设综合实现程度达83%，比2003年提高18.7个百分点。中心城市综合实力全国百强排名从2002年83位跃升到2006年39位。统筹城乡发展成效显著，县域经济活力增强、发展加快，占全市经济总量比重提高近10个百分点，四县全部进入全国最具投资潜力中小城市百强。

坚持把三农工作摆在重中之重的位置，认真落实扶农支农政策，加快农业产业结构调整，农村经济全面发展。积极推进高效农业规模化，高效农业面积达123万亩，海水工厂化养殖38万平方米，畜禽规模化养殖比重提高10个百分点，农业机械化水平达69%，粮食生产连续四年丰产丰收。农业产业化进程加快，发展龙头企业基地166.3万亩，优质稻米、特色渔业、出口蔬菜等特色产业不断壮大，省级以上重点龙头企业增至17家，农民专业合作组织达264家。外向型农业快速发展，形成蔬菜、水产品等20个规模化出口基地，农产品出口跃居全省第一。加快农业生产标准化建设，大力发展生态农业。建成省以上无公害农产品标准化示范区12个，无公害、绿色、有机食品品牌230个，成为全省第一家无公害农产品产地整体推进市，农产品质量明显提高。

坚持工业第一方略，加快新型工业化步伐，工业经济主体地位不断提升，2007年增加值占地区生产总值比重较2002年提高7.9个百分点。工业布局进一步优化，各类产业园区加快基础设施建设和项目入驻，市临港产业区、灌河半岛经济区和灌云、赣榆、连云等县区沿海开发快速启动，东海、新浦、海州产业园区发展空间进一步拓展，沿海和沿东陇海线产业带框架初步形成。新医药、新材料、新能源产业竞争力明显增强，高新技术产业产值年均递增36.4%，占规模以上工业比重提高9.8个百分点。食品、化工等传统产业改造提升步伐加快。临港工业发展迅速，船舶修造、汽车制造等现代装备制造业项目取得突破。规模以上企业突破1000户，增加值占全部工业比重达83.6%，提高24个百分点。田湾核电站一期工程投入商业运行，阳光晶源硅晶体材料、中复连众风机叶片等重点项目建成投产，万吨级碳纤维项目开工建设，新电机组扩建等25个国家技改项目顺利实施，恒瑞医药、鹰游纺机等4户企业入选省重点培育发展的百强企业。加强质量品牌建设，培育出长江三角牌纯碱等中国名牌4个、连利牌水表等国家免检产品7个，东海水晶获国家地理标志产品保护，企业核心竞争力和产业素质不断提高。建筑业增加值年均递增16.4%，外出建筑务工294.6万人、实现产值506亿元。

坚持把发展现代服务业作为优化结构的重要途径，依托优势，打造特色，支撑和服务区域发展的功能进一步增强。中云物流园区、陆桥保税库等重点项目建设进展顺利，全国首家专业棉花保税库建成投运，引进一批第三方物流龙头企业，口岸查验单位强化服务和监管，以港口为中心的现代物流体系不断完善。开通至莫斯科集装箱五定班列，大陆桥运输实现欧亚贯通。开辟欧洲、美国等远洋干线，至韩国仁川、平泽客货班轮航线开通营运。港口吞吐量连跨5个千万吨台阶，2007年达8506.8万吨，集装箱运量突破200万标箱，跻身集装箱运输沿海十大港、全球百强港。民航航线增加到10条，客运量达21.6万人次。以山海风光为特色的旅游业加快发展，成功创建中国优秀旅游城市，新增国家AAAA级景区3个、国家工农业旅游示范点10个，举办旅游系列节庆活动，在中央电视台等主流媒体开展城市形象宣传，境内外游客接待量、旅游总收入年均递增18%和23%，被国家旅游局评为全国旅游业发展最快的三个地级市和全国20个优秀旅游目的地之一。大陆桥国际商务中心列入省首批服务业集聚区。连锁经营、品牌专卖逐步成为城镇商贸主要业态，新建改建农家店1000家，城乡消费市场活跃，价格监管得到加强。2007年实现社会消费品零售总额247.5亿元，比2002年翻一番。金融机构有效服务经济建设，银企、银政合作进一步加强。房地产、社区服务等新兴服务业加快发展。

二、深化体制机制创新，改革开放实现新突破

各项改革逐步深化。不断完善行政管理体制，调整强化安全生产、食品药品监管及公共服务等职能，清理精简行政审批事项，实施行政区划调整。市属生产经营型事业单位改制转企，实施首批141家市属社会公益型事业单位人事和分配制度改革。市属国有工业企业、非工业流通企业改制基本完成，资产重组9.6亿元，妥善安置破产企业职工和离退休人员3.8万人。国有资产监管体制改革有序推进，组建连云发展集团、东方资产管理公司、金港湾投资公司等资本运营主体，市县区国有资产市场化运作体系基本建成。加快投融资体制改革，连云港股份上市融资，港口集团企业债券成功发行，运用投资与建设组合模式推进疏港道路、物流园区等项目建设。金融体制改革稳步推进，城市商业银行重组并入江苏银行，组建东方农村合作银行，金海担保公司重组增资。积极构建公共财政框架，完善部门预算、国库集中支付、政府采购制度和市对区财政管理体制。农村综合改革扎实推进，落实免除农业税政策，开展农业保险试点。市场取向的改革和政策导向的激励有效激发民营经济活力，成为开辟税源、吸纳就业、推动发展的重要增长点。2007年民营经济增加值占地区生产总值51.9%，比2002年提高18.4个百分点。

对外开放成效明显。深入实施开放兴市战略，强力推进招商引资。累计注册外资实际到账超过17亿美元，占改革开放以来总和的69%。内联资金到位728.6亿元，年均递增64%。引进德国汉高、青年汽车等一批国内外知名企业。成功承办举办江苏国际农洽会、苏台经贸洽谈会等经贸活动。对外贸易持续增长，出口结构进一步优化。累计完成出口55.7亿美元，年均递增29.7%，高新技术产品出口规模不断扩大，机电产品出口比重提高11个百分点。对外经济合作领域进一步拓宽，4家企业获商务部对外承包工程和劳务合作经营资格，新办境外企业24家，累计完成外经营业额8.5亿美元，年均递增20%。连云港经济技术开发区开放发展步伐加快，在53个国家级开发区发展水平综合评价中位次前移，出口加工区获批运作，新增省级开发区5个，各类开发园区载体功能和集聚效应显著增强。

三、着力完善基础设施，城乡面貌展现新形象

坚持规划引领建设。组织战略研讨，面向国际招标，编制东部滨海地区发展战略规划，修编城市总体规划，组织编制30多个专项规划，确立一心三极海滨城市、一体两翼组合大港、一纵一横T型产业走廊的发展布局，中心城区控制性详规覆盖率达100%，东区规划展览馆建成开放。累计投入城市建设资金250亿元，建成区面积扩大到90平方公里，城市化水平达40%以上。强力推进东部城区建设，实施海滨新区陆域形成工程，中央商务区、连岛等节点开发全面启动，建成大陆桥国际商务大厦等一批标志性建筑，海滨城市风貌初步显现。新海城区空间拓展、功能提升、形象刷新，凤凰、东河、孔望山片区启动建设，旧城改造步伐加快，城区西、南出入口整治基本完成。开工建设花果山大道，新建改建港城大道、海宁大道、中山路等147条城市主次干道，城市路网进一步完善。房地产竣工780万平方米，其中住宅536万平方米。建成郁洲公园等98个公园广场和游园绿地，新增城市绿地1390公顷，城市绿化覆盖率达37.5%，成功创建省级园林城市。新增人防工程25万平方米，建成市人防基本指挥所和民防应急指挥中心。供水、排水、燃气、供热、环卫等公用事业快速发展。加强城市精细化管理，市容环境明显改观。四县县城建设步伐加快，集聚辐射功能进一步增强。培育发展一批省新型示范小城镇、重点中心镇。

基础设施建设加快推进。累计完成港口建设投资72.9亿元，实施15万吨级航道扩建、25万吨级矿石码头、庙三突堤集装箱码头、疏港通道等一批重点工程，新增吞吐能力1800万吨，服务区域经济发展的功能显著提升。累计完成交通建设投资104亿元，连霍、沿海高速公路连云港段建成通车，连临高速公路开工建设，高速公路通车里程达288公里。新建改建国省道干线公路382.8公里，第一轮网化工程基本完成。通榆河北延工程、盐灌船闸开工建设，204国道新沂河特大桥、海堤公路建设加快推进。基本完成东陇海铁路电气化改造，中云火车站开工，连盐铁路列入国家十一五路网规划。民航投入力度加大，顺利通过国家安全审计。以港口为中心的立体交通网络初步形成，成为交通部规划建设的长三角地区7个国家级综合运输枢纽之一。实施新沂河整治和小塔山、安峰山水库除险加固等一批重点水利工程，在省内率先完成海堤达标建设任务。改造城乡电网，伊芦、蔷薇等一批输变电工程建成投运。通信网络不断完善，信息化建设步伐进一步加快。

四、推动社会事业进步，协调发展取得新成效

科技教育优先发展。大力实施创新驱动战略，推动技术进步和科技成果转化，区域发展竞争力不断提升。实施国家863计划项目16项，省科技成果转化资金项目13项，省以上火炬、星火计划项目372项，新认定国家重点高新技术企业9家、省高新技术企业49家，建成新医药、硅材料国家火炬计划特色产业基地和汉高华威、恒瑞医药国家863计划成果产业化基地，三新产业、现代装备制造等重点领域自主创新取得突破，科技先导型区域支柱产业基本形成，被列为省十一五制造业信息化示范城市。设立国家认定企业技术中心2家、省级工程中心12家、博士后科研工作站6个，专利授权量达916件。加强与清华大学、中科院等单位合作，组建江苏省海洋资源开发研究院，产学研对接成效明显。推进教育均衡发展，免除义务教育阶段学杂费，新扩建校舍98万平方米，城乡基础教育办学条件和教育质量进一步改善，小学、初中入学率分别达99.99%和99.91%，高中段入学率达92%。创成省四星级高中4所、国家级重点职业学校6所。高等教育在校生达4.2万人，科教创业园区开工建设。建立省级引智成果示范推广基地4个。引进本科以上学历毕业生和高层次人才15213人。

各项社会事业协调发展。累计用于社会事业发展的财政支出62.7亿元，是前五年的2.1倍。市紧急救援中心、中医院门急诊楼、少儿活动中心、气象防灾减灾中心等一批社会事业重点项目建成启用，市体育中心、妇女活动中心、传染病医院、第一和第二人民医院综合楼等项目开工建设。文化建设繁荣有序，516件文艺作品获省以上奖项，在海一方系列活动获全国特色文化广场活动称号，在北京人民大会堂成功举办连云港儿女唱响和谐中国大型主旋律音乐会。新增藤花落遗址、海清寺塔2处国家级和5处省级文物保护单位，海州五大宫调入选全国第一批非物质文化遗产名录，建成市博物馆新馆、文化艺术中心等标志性文化工程。有线电视入户率53%，行政村联网率100%。宽带网络覆盖所有行政村。成功承办世界沙滩排球巡回赛、十运会部分项目等省级以上赛事25项，获省以上竞技体育比赛冠军182个，其中世界冠军4个。全市乡镇、社区和行政村全部建有体育健身设施，全民健身运动蓬勃开展。启动和谐奥运·微笑连云港系列活动。城市社区卫生服务实现以街道为单位全覆盖，新型农村合作医疗参合率达96.5%。成功抗击非典，公共卫生应急体系进一步完善，传染病发病率控制在全省平均水平以下。人口和计划生育管理服务不断加强，建立健全利益导向机制，出生人口性别比升高势头得到有效遏制，人口自然增长率保持在5‰左右。国防建设、民兵预备役和优抚安置工作得到加强，连续五次被命名为全国双拥模范城。妇女儿童工作受到国家表彰，成功创建国家级防震减灾科普教育基地，市档案馆建成江苏省一级档案馆。完成第一次经济普查，开展市、县两级全面小康社会建设监测统计。外事、侨务、民族宗教、气象、地方志、老区开发工作取得新成绩，老龄、关心下一代及残疾人事业得到重视和发展。

精神文明和民主法制建设扎实推进。加强社会主义荣辱观教育，推行公民道德规范，推进文明城市创建，城乡共建文化先进村，城市文明形象和市民文明素质明显提升。邓小平和人民在一起群雕落成，抗日山被命名为全国爱国主义教育示范基地，陇海步行街被授予全国百城万店无假货示范街称号，新浦汽车客运总站被命名为全国雷锋精神教育基地。定期向人大及其常委会报告工作，向政协通报情况，注意听取各民主党派、工商联和各人民团体意见。累计办理人大代表建议874件、政协委员提案1578件，代表、委员满意和基本满意率在97%以上。依法行政制度建设不断强化，建成电子政务平台，行政效能明显提高。加强基层民主政治建设，村民和社区居民自治有序推进。开展领导干部下访活动，强化信访基础工作，畅通群众利益诉求渠道。普法工作深入推进，法律援助实现应援尽援，社会矛盾大调解格局基本形成。健全应急管理体系，完善社会治安防控网络，群众安全感和社会治安满意度不断提高，被评为全省社会治安安全市和综合治理先进市。认真落实廉洁从政各项规定，不断完善述职述廉、民主评议和重大事项报告等制度，治理商业贿赂，规范教育、医疗和涉农、涉企等收费行为，强化行政监察和审计监督，廉政建设得到加强。

五、切实改善群众生活，社会和谐呈现新局面

努力提高人民生活水平。坚持富民优先，多渠道增加群众收入，建立企业职工工资随效益增长机制，提高最低工资标准。2007年，城市居民人均可支配收入13254元，农民人均纯收入4740元，年均分别递增13.8%和9.6%。五年归集城镇职工住房公积金27.2亿元，城镇人均住房建筑面积由27.4平方米增加到31.2平方米，农村人均钢筋砖木结构住房面积由21.9平方米增加到27平方米。居民消费层次不断提升、结构逐步优化，休闲度假、网络服务、家庭汽车、住房等新型消费和大宗消费迅速发展。

切实加强就业和社会保障。认真落实积极的就业政策，多渠道开发就业岗位，累计实现就业再就业19万人，城镇登记失业率由2002年4.3%下降到3.1%。采取政府购买岗位等援助措施，帮助4.2万人实现就业，零就业家庭动态为零。加快转移农村劳动力，实现劳务输出101万人。累计用于社会保障的财政资金16.6亿元，年均递增29.9%。养老、医疗、失业、工伤、生育等社会保险覆盖面不断扩大，城镇居民基本医疗保险启动实施，在全省率先将农民工纳入城镇企业养老保险范围。积极构建和谐劳动关系，切实保障劳动者合法权益。城乡低保实现全覆盖，44万人次低保对象基本生活得到保障，农村五保对象集中供养率达54.6%。完善城乡低保标准自然增长机制和自然灾害应急体系，建立对家庭经济困难大学新生的助学机制，健全经常性社会捐助制度，慈善事业得到社会各界的关心和支持。

扎实兴办利民实事。五年来，新建改建城区便民道路190条，更新人行道板260万平方米，新装小街巷路灯3000套，新建改建公厕85座，开通优化新浦至东部城区等公交线路，增建城市公交站场，更新公交车辆，建设经济适用房27万平方米，解决2620户家庭住房难问题，市县城镇最低收入住房困难家庭全部纳入廉租住房保障范围，居民出行居住条件明显改善。扎实推进农村七件实事和新五件实事，建成农村公路6200公里，所有乡镇、99%行政村通水泥或柏油路，行政村客运班车通达率96%，改水普及率96.8%，改造草危房4万户，完成40个乡镇文化站达标建设，加强农村实用技术和劳动力就业培训，实施农业综合开发，组织百名教授科教兴百村、民企帮村、联动帮扶等支农活动，有效带动农民增收。完成全市镇村布局规划、村庄整治建设规划，实现市域乡村规划全覆盖。通过规划引导、典型示范、项目化推进，社会主义新农村建设取得初步成效。

着力促进和谐发展。坚持环保优先、节约优先，狠抓节能减排、污染防治、饮用水源保护，扎实推进生态保护与建设。大力发展循环经济，开展小化工、小化肥、采石塘口等专项整治，实施云台山封山育林，排污总量得到有效控制，森林覆盖率提高6个百分点，空气质量优良率在90%以上，声环境优于国家标准。国家环保模范城市创建工作通过国家技术评估，东海、赣榆生态示范区创建工作通过国家考核验收，四县全部被评为全国绿化模范县。落实耕地保护政策，坚持集约利用土地，国土资源管理和土地复垦开发得到加强。坚持科学用海、合理开发，海域管理水平全国领先。深入整治各类安全隐患，健全责任体系，强化基础工作，连续五年实现安全生产事故起数、死亡人数双下降，连续三年获得省政府安全生产目标考核一等奖。

各位代表，过去的五年，全市各项工作取得了可喜成绩。特别是刚刚过去的2007年，是我市发展进程中具有特殊意义的一年，中央、省赋予连云港更高的发展定位、给予更大的关心和支持，连云港迎来前所未有的重大机遇。这一年，全市经济社会发展保持十一五首战之年的良好势头，地区生产总值增长15.1%，财政总收入跨上百亿元台阶，县域经济发展实力快速提升，各项事关全局、事关长远的重点工作扎实有效推进，全市又好又快的发展态势进一步显现;城乡居民收入迅速增加，改革发展成果共享度不断提高，群众生活明显改善，人心思进、创业发展的氛围更加浓厚，构建和谐社会的基础进一步强化;开放的港城受到海内外有识之士的广泛关注，各类资源加快汇聚，实际到账注册外资7亿美元、比上年翻一番，总量、增幅分别居苏北、全省前列，区域发展综合优势进一步彰显;国际性海滨城市建设框架全面拉开，组合港建设步伐加快，产业发展加速推进，城市、港口、产业互促联动发展效应进一步增强，规划绘就的美好愿景正快步走向现实。

回顾过去五年的工作，我们有以下几个方面的体会：

一是必须狠抓第一要务，积极探索符合科学发展观要求、有地方特点的发展道路。立足对我市基本条件、主要矛盾、战略机遇、发展阶段的分析判断，不断完善发展思路，确立以工兴港、以港兴市、以市带农发展方针，明确城市、港口、产业布局框架。坚持既要加快发展、跨越发展，又要科学发展、和谐发展;坚持以高水平规划统筹当前与长远，统筹城乡区域发展;坚持生产发展、生活富裕、生态文明并重，全面落实行之有效的推进措施，全市发展逐步驶入良性运行的快车道。从加快发展上升到科学发展，在跨越发展中追求更好更快，对于全面奔小康，重振连云港将起到决定性作用。

二是必须不断解放思想，切实通过改革创新有效破解发展难题。坚持以思想解放消除观念制约、体制障碍，在不断深化各项改革的同时，针对发展中的困难和问题，积极转变观念，创新思维，主动探求解决办法。几年来，成立若干投融资主体，将潜在资源整合成项目，通过国际国内资本市场运作，有效缓解资金瓶颈;通过争取盐场管理权下放，扩充了发展必需的土地资源;通过争取使我市成为全国海域使用开发首家试点城市，为海滨新区、港口建设及旅游发展预留了用海空间。实践证明，改革创新可以不断增强发展活力，能够有效拓展发展空间，也必将为持续发展注入不竭动力。

三是必须坚持发展靠开放，广泛集聚各类发展资源。立足和放大区位、交通、能源、土地、环境等综合优势，努力在更大范围、更高层面集聚资源，有力推动和支撑发展。认真落实温家宝总理来连重要指示和省委、省政府重要部署，主动对接，跟进落实，使连云港在全国、全省的战略地位得到进一步确认和提升。港口管理体制改革后，以建立现代企业制度为方向组建集团公司，积极引进中海、中远等战略合作伙伴，多元投入加快建设发展，成为交通部规划建设的全国沿海主要港口和长三角港口群三大主体港之一，为建成集装箱优先发展的亿吨大港、干线大港奠定了坚实基础。积极推进与周边地区和陆桥沿线重点城市合作，区域携手发展、互利共赢空间日益广阔。

四是必须更加重视改善民生，使广大群众共享改革发展成果。本届政府就任之初，明确提出坚决不要损害群众利益的政绩，不要以牺牲环境为代价的政绩，努力善待群众、厚待群众。坚持把发展经济与造福百姓结合起来，把促进经济增长与扩大就业协调起来，把先富群体和困难群体的利益兼顾起来，把改善群众生活与促进社会和谐统一起来，认真解决群众最关心、最直接、最现实的利益问题，真正使发展为了人民、发展依靠人民、发展成果由人民共享成为政府的行动指南。

回顾五年历程，我们深深感到，全市改革发展成就的取得，得益于中央、省一系列方针政策的科学指引，是480万港城人民在市委正确领导下团结奋斗的结果，是与历届市政府打下的良好基础和市人大、市政协的监督支持分不开的。在此，我代表市人民政府，向全市广大干部群众、驻连部队、武警官兵、公安干警、驻连部省属单位和支持连云港发展的海内外朋友，向各位人大代表、政协委员和各民主党派、工商联、各人民团体、各界友好人士，表示崇高的敬意和衷心的感谢!

在肯定成绩的同时，必须充分认识全市发展中存在的矛盾和问题，主要是：在日趋激烈的区域发展竞争中，综合实力还不强，带动作用明显的重大产业项目不多;产业结构层次还不高，节能减排的硬约束日益强化，转变经济发展方式的任务紧迫;城乡发展不平衡，新农村建设基础薄弱，农民收入持续快速增长的难度较大;当前物价上涨给城乡居民生活带来影响，群众在就业、住房等方面还有许多新期待;政府机关服务水平、办事效率有待进一步提高。我们必须增强忧患意识、责任意识，高度重视并及时化解前进道路上的各种困难和问题，努力把各项工作做得更好。

　今后五年展望和2008年工作建议

党的十七大确定了继续全面建设小康社会、加快推进社会主义现代化的战略部署，胡锦涛总书记、温家宝总理对连云港的发展寄予殷切期望;省委十一届三次全会进一步丰富完善全面达小康、建设新江苏内涵，提出全面建设更高水平小康社会的新要求。这为连云港在新起点上实现更好更快发展，指明了前进方向，提供了巨大动力。从现在到2012年的新一届政府任期，是连云港全面建设小康社会的关键时期，是衔接两个五年发展规划，承前启后、继往开来的重要阶段。我们要根据中央、省对连云港的发展定位、工作要求和市第十次党代会部署，牢牢把握发展第一要务，抢抓机遇第一资源，继续解放思想，坚持改革开放，推动科学发展，促进社会和谐，建设国际性海滨城市、现代化港口工业城市和山海相拥的知名旅游城市，加快成为苏北振兴、沿东陇海线和江苏沿海开发的龙头，充分发挥连接南北、沟通东西的重要枢纽作用。努力在以下几个方面取得重大进展：

--转变发展方式，促进又好又快发展。把产业结构优化作为基本途径，把增强创新能力作为核心环节，把节能减排作为重要突破，切实转变发展方式，在提高质量效益和优化经济结构的基础上保持较快增长。重点发展三新产业、现代装备制造、现代物流、旅游等先进制造业和现代服务业，建设新型临港产业基地，加快成为区域发展重要增长极。地区生产总值比2007年翻一番，三次产业结构调整为9:53:38。财政一般预算收入年均增长25%，比2007年增长2倍。

--注重改善民生，推动社会和谐进步。加快富民步伐，城市居民人均可支配收入年均递增10%，农民人均纯收入年均递增8%以上;落实城乡居民低保标准增长机制，按照每人每天生活费不低于1美元的国际标准，全面消除绝对贫困现象;以学有所教、劳有所得、病有所医、老有所养、住有所居为重点，加快发展社会事业，提高基本公共服务均等化程度;推动文化发展繁荣，切实保障人民群众在经济、政治、文化等各方面的权益，促进社会公平正义;注重节约资源、保护环境，努力营造生态文明、社会进步、百姓安居乐业的和谐新局面。全面小康社会建设综合实现程度达98%左右。

--深化改革开放，增强发展活力和动力。广泛集聚发展资源，推动利用外资扩大规模、优化结构、提高质量和效益，注重招商引智，加快港口共建共用、陆桥沿线联合开发，努力打造开放型经济新高地。创新体制机制，加大重点领域和关键环节改革攻坚力度，争取国家改革创新政策支持，增创竞争发展优势。实际到账注册外资、出口年均分别递增20%和16%。港口吞吐量达1.4亿吨、集装箱运量500万标箱。连云港经济技术开发区在国家级开发区中位次前移。建成区域性商务中心。

--建设海滨城市，加快城市化和城市现代化进程。以国际水准规划体系为引领，强化基础设施建设，完善城市功能，提升城市品质，推动城区组团融合联动发展，加快建设便捷、通畅、高效、安全的现代综合交通运输体系，初步建成辐射带动力强、充满活力、富有魅力、适宜人居的现代化国际性海滨城市。中心城市建成区面积150平方公里，城市化水平达52%。

--发展县域经济，建设社会主义新农村。以发展生产为中心，以农民增收为核心，加强农业基础建设，繁荣农村经济。以工促农、以城带乡，在发展规划、产业布局、基础设施、公共服务、劳动就业、社会管理等方面加强城乡统筹，促进一体化发展，富裕文明和谐新农村景象进一步展现。

各位代表，2008年是党的十七大胜利召开后的第一年，是新一届政府的开局之年，也是实现十一五发展目标的重要一年。根据中央、省经济工作会议和市委十届五次全会部署，今年政府工作总体要求是：全面贯彻党的十七大精神，以邓小平理论和三个代表重要思想为指导，深入贯彻落实科学发展观，自觉服从和主动顺应宏观调控，坚持好字优先、好中求快、优中求进，突出主攻项目，夯实基础，全面提升，加快跨越，进一步解放思想、抢抓机遇，加快改革创新和对外开放，加快转变发展方式，重抓经济结构优化和节能减排，重抓海滨城市建设和农民增收，重抓民生改善和社会建设，又好又快推进全面奔小康，重振连云港，努力开创科学发展、率先发展、跨越发展、和谐发展新局面。

今年经济社会发展主要预期目标是：地区生产总值增长15%，节能减排完成省下达任务，财政一般预算收入增长25%，全社会固定资产投资增长25%，社会消费品零售总额增长16.5%，实际到账注册外资8亿美元，出口增长15%，城市居民人均可支配收入、农民人均纯收入分别增长10%和9%，居民消费价格涨幅不高于全省平均水平，城镇登记失业率低于3.4%，城市化水平提高2个百分点。

全市着力抓好十项重点工程：(1)庙三突堤5个集装箱码头建成使用，25万吨级矿石码头完成主体工程。(2)通榆河北延工程实施全线征地拆迁、河道开挖和灌北泵站建设;疏港航道全线开工，建设新沂河对口船闸。(3)204国道新沂河大桥建成通车，盐灌船闸完成主体工程。(4)海滨新区完成海堤抛填工程，建成东方国际商务中心，启动东方商务广场建设，完成胜利湖景观改造工程。(5)花果山大道全面建成，完成海棠路立交桥主体工程。(6)建成中云火车站，完成东部城区公交总站主体工程，开工建设客运码头，扩建民航国际候机厅。(7)完成新沂河整治主体工程，开工建设新沭河50年一遇治理工程。(8)大浦河调尾工程完成5公里河道开挖。(9)完成市体育中心体育馆土建工程。(10)师专和职业技术学院新校区投入使用。

全市认真做好十件民生实事：(1)经济适用房竣工13万平方米，为1240户低收入家庭提供廉租房保障。(2)建设便民道路60条，整治人行道板5万平方米，新建农村公路1000公里、客运站10个，改造农村公路危桥30座。(3)新增5万农民参加新型农村社会养老保险，农村五保户集中供养率提高到60%;建设市社会福利中心和市老年大学，落实老年福利养老机构床位补贴。(4)市区饮用水源地水质稳定达标，进一步实施农村饮水安全工程。(5)新建100个农村社区卫生服务站。(6)新建市农副产品批发市场，改造规范农贸市场10个，新建日用消费品和农资连锁农家店300家。(7)药品生产经营使用环节监督率达100%，药品零售企业远程监控率达50%，行政村食品放心店覆盖面达80%。(8)有线电视入户率提高到60%，建成24个乡镇文化站。(9)开通农业科技人工语音服务热线。(10)社会治安实现科技防控全覆盖，建立100个乡镇、街道法律援助站。

今年着重抓好以下六个方面工作：

一、以产业结构优化为主线，加快推进新型工业化

积极调整产业结构，持续扩大有效投入，加快有地方特色的新型工业化进程，促进工业经济总量和质态同步提升。一是突破临港工业，以新型主导产业促进产业升级。着力推进船舶修造等大型项目的建设。二是大力发展新医药、新材料、新能源等优势高新技术产业，积极培育和壮大纺织机械、物流装备、汽车及零部件等现代装备制造业，以产业集群推动结构优化。加快实施中复神鹰碳纤维、纺织工业园、盛大压力容器等120个投资过亿元项目，高新技术产业产值增长30%。

三是提高自主创新能力，加强信息化带动，提升传统产业技术水平，以技术进步驱动产业升级。实施重点自主创新和产学研项目200个。推进知识产权战略，申请专利850件。引导企业采用国际标准和国外先进标准20项，争创中国名牌2个、国家免检产品2个、省名牌10个。四是聚集要素资源，加强扶持引导，培植骨干企业支撑产业结构提升。新增规模工业企业100户，销售收入超亿元工业企业达120户，超10亿元企业12户。五是强化规划引导，促进企业向临港沿线集中，依托园区载体调整产业布局。沿东陇海线和所有临近城区工业园区以发展都市型加工制造业为主。省级以上园区规模工业销售收入占比提高2个百分点。严格控制小化工等高污染高耗能行业发展，淘汰落后工艺与设备，加快重点能耗企业节能改造，推动能耗水平进一步下降。建筑业增加值增长16%以上。

适应新型工业化和居民消费结构升级要求，优先发展生产服务业，规范提升生活服务业，促进服务业规模扩张、结构改善、功能优化。大力发展现代港口物流，推进金港湾国际物流园等基础设施建设，增开远洋干线和内贸航线12条以上，加快大陆桥集装箱运输为主导的区域性国际物流中心建设步伐。积极发展科技服务业，建立健全自主创新和社会化科技服务体系，搭建科技研发创新平台，建设市科技创业服务中心，用好开发区、金海浦南等科技企业孵化器。大力发展代理、咨询、租赁等商务服务业，优化金融生态环境，引导金融服务创新。加快知名旅游城市建设，注重打造特色、提高品质，完善配套设施和服务网络，创新旅游管理体制和开发机制，扩大旅游资源知名度、共享度，国内外游客接待量、旅游总收入分别增长15%和16%。引进扶持知名品牌，运用现代经营理念和信息技术，改造提升商贸餐饮等传统服务业。着力发展面向民生的物业、社区、家政等生活服务业，积极发展适应消费需求的文化、体育、休闲等新兴服务业，拓展领域，提升层次，培育服务业新的增长点。强化政策扶持和人才引进培养，大力发展以承接服务外包为重点的国际服务贸易。着力抓好国际商城改造、新海粮食物流中心等16个重点项目建设，完成投资23.6亿元。服务业增加值增长16%。

二、以增加农民收入为核心，扎实推进新农村建设

加快发展高效生态外向农业。深入推进结构调整，推动高效农业规模化。大力发展设施农业，新增高效农业种植面积20万亩、海水工厂化养殖2万平方米，畜禽规模化养殖比重提高2个百分点。扶持壮大龙头企业，畅通农产品流通渠道，提升农业产业化水平。新增市级以上重点龙头企业5家，省级以上重点龙头企业销售收入增长16%，新增农民专业合作组织30家。加强农畜产品质量监测，推进农业标准化建设，新创无公害、绿色、有机食品品牌50个，申报省级以上农业标准化示范区2个，加强高致病性禽流感等重大动物疫病防控，保障农产品质量安全。发展壮大20个农产品出口基地和20家重点出口企业，培植市场主体，实施农业外向化拓展，农产品出口增长20%。

切实加大强农惠农力度。全面落实涉农补贴政策，整合各项支农资金，积极引导信贷资金、社会资金和外资，多元投入新农村建设。深入开展送科技下乡、百名教授科教兴百村等科技支农活动，重点推广200项农业新品种、新技术，主要农作物机械化水平提高到71%。完成农业综合开发8500万元，改造中低产田10万亩。扎实推进政策性农业保险试点。加强农村劳动力就业培训，完成农村实用技术培训50万人次，新增转移农村劳动力培训率达60%以上。深入推进挂钩帮扶，实现218个经济薄弱村1/5的低收入人口脱贫。

着力改善农村生产生活条件。完成30%以上规模较大、历史文化遗存丰厚、地形地貌复杂三类村庄的建设规划，稳步推进新农村规划居住示范点建设。实施农村道路桥梁建设改造，行政村客运班车通达率达98%。加快信息化村镇建设，提高农村信息化应用水平。综合整治农村环境，重点推进清洁水源、清洁家园、清洁田园和绿化造林，开展县乡河道和村庄河塘疏浚，发展沼气用户1万户，推动改圈、改厕、改厨，农村地表水环境质量达标率提高3个百分点。

三、以体制机制创新为动力，大力推进改革开放

创新利用外资方式，积极推动招商选资。着眼延伸产业链、构建产业集群、优化产业结构，搞好各类招商活动，重点突破一批投资规模大、带动能力强、综合效益高的项目。承接国内外大企业集团产业转移，对接区域产业政策、产业规划，引导外资投向先进制造业、现代服务业、现代农业等重点领域，着力引办一批高新型、生态型、节约型项目。大力推动专业化、精细化招商和以商引商，突出修造船、汽车、石化、现代物流、硅材料等重点行业，加强重大项目开发储备，围绕天合光能多晶硅、华磁商用电器等一批拟建、在建重点项目，强化跟踪服务，优化投资环境，提高项目签约率、资金到位率、开工投产率。以国家级开发区、临港沿线产业园区为重点，加大基础设施投入，完善配套服务，加强功能创新、科技创新、管理创新，推进南北共建，打造布局合理、特色鲜明、产业集聚、生态和服务环境优良的园区体系，进一步提高产出效能。优化出口产品结构，扩大机电和高新技术产品出口，促进加工贸易转型升级。提升外经合作层次，加快新医药、新材料等支柱行业企业走出去步伐。

推进投融资体制改革，鼓励企业运用上市、股权融资、并购重组、发行债券等方式集聚发展要素，促进连云发展集团、港口集团等国有资本运营主体快速扩张。健全国有资产管理体制，建立国有企业经营预算管理制度，完善风险控制、保值增值、经营者奖惩考核等各项监管制度。推进事业单位改革及配套改革，完善财政管理体制，稳步推进农村综合改革。健全中小企业融资担保、科技服务等社会化服务体系，支持中小企业提高管理水平和创新能力，深入实施微小企业成长计划，扶持做大做强一批重点骨干民营企业，民营经济增加值增长16%。

四、以实现亿吨大港为突破，加速推进海滨城市建设

坚持以港兴市，加快以港口为龙头的基础设施建设，推动港口扩能上量，增强对城市发展的带动能力。主体港区完成15万吨级航道扩建等重点工程，深化30万吨级航道、大堤作业区前期工作。加快两翼港区规划建设，完工中交三航局、恒成船业等专用码头，推进灌河口拦门沙治理和航道工程研究。完善港口集疏运体系，基本建成南疏港通道，加快东疏港高速公路建设，北疏港高速公路开工。完善口岸大通关，建设公共信息化平台，实施口岸联检，建立港口检验检测基地，进一步提升服务功能。港口吞吐量突破1亿吨，集装箱运量达300万标箱。年内新增2条民航航线，推进空港对外开放。加快连临高速公路建设，新改建310、226、242等国省道干线。推进龙河输变电、宽带传输扩容、邮政枢纽等电力、通信基础设施建设。

加快国际性海滨城市建设，提升对港口、产业的服务承载功能。突出城市特色，进一步完善规划成果，开展景观轴线、视觉廊道规划。新建续建商务核心区山海景观轴、中央景观大道，加快凤凰、东河、孔望山片区开发，建设金海国际大厦、金港湾商务大厦等标志性建筑，实施南极北路、外仓街等集中成片危旧房改造。加快市妇女活动中心、市第一人民医院综合楼等社会事业重点项目建设，第二人民医院综合楼投入使用。新建改建郁州南路、平山北路等30条城市主次干道，进一步优化路网结构。优先发展城市公共交通，规划建设各类停车场地。新增人防工程8万平方米。新建改建自来水干线20公里，新增管道天然气用户8000户、集中供热50万平方米，完成城区污水管网15公里，墟沟污水处理厂正式投入运行，建成东海县尾水通道工程。推行物业纳入社区建设的新模式，推进市容精细化管理，加快环卫作业市场化步伐，实施一批美化亮化精品工程，新增城市绿地220公顷。推进国家卫生城市、国家环保模范城市、国家园林城市、省文明城市创建工作。进一步提高县城规划建设管理水平，加快重点中心镇、特色名镇建设，开展新型小城镇、康居示范村创建，推动县城、城镇和农村协调发展。

五、以改善民生为重点，务实推进和谐社会建设

切实抓好就业和社会保障。实施积极的就业政策，支持发展自主就业，培育更多创业主体，促进以创业带动就业。加大就业培训力度，积极开发公益性岗位和社区服务岗位，完善面向所有困难群众的就业援助制度。建立城乡统一的就业和失业登记管理制度，逐步把城镇就业再就业政策拓展到农村劳动者。新增就业和再就业4.6万人，新转移农村劳动力5万人。健全职工工资正常增长机制，在岗职工平均工资增长10%以上。完善城乡居民社会保障体系，建立农村新型养老保险制度，城镇居民基本医疗保险参保率达90%，农民工医疗和工伤保险基本实现全覆盖，被征地农民、失海渔民、失田盐工基本生活做到即征即保。扩大廉租住房保障范围，建设经济适用住房27万平方米，进一步改善城市中低收入家庭住房条件，继续实施人才安置房工程，推进解决城市农民工和外来人员住房问题。加快构建以最低生活保障、五保供养、特困救助、临时救济、医疗救助为主要内容的城乡社会救助体系，帮助困难群众、低收入群体解决实际困难。鼓励引导专项和经常性捐赠活动，促进社会福利事业多元化发展。重视发展老龄事业，启动社会养老工程。充分调动各方面力量和资源，让农村留守流动儿童得到更多关爱。

统筹发展各项社会事业。实施基础教育提高工程，促进义务教育均衡发展，小学、初中入学率保持在99.9%以上。加快普及高中阶段教育，大力发展职业教育，积极提升高等教育。推动公共教育资源向农村倾斜，提升农村教育教学质量和办学水平，新扩建农村中小学校舍10万平方米。在全面免除义务教育学杂费基础上，实行城乡义务教育全部免费提供课本。健全教育资助制度和助学体系，保障经济困难家庭学生、进城务工人员子女平等接受义务教育。创新人才工作机制，加大人才开发力度，引进本科以上学历毕业生3000人。优先发展公共卫生、农村卫生和社区卫生。加快公共卫生体系建设，增强疾病预防控制和应急救治能力。完善农村三级卫生服务网络，新型农村合作医疗参合率巩固在95%以上，城市社区卫生服务人口覆盖率达90%以上。积极应对第四次生育高峰，稳定低生育水平，提高出生人口素质，开展省十一五人口协调发展先进县区创建，计划生育率达90%以上，人口自然增长率控制在7‰以内。加强国防动员和后备力量建设，提高优抚安置保障水平，扎实推进双拥工作。组织开展第二次经济普查。继续做好外事、侨务、对台事务、气象、防震减灾、老区开发等工作，支持发展妇女儿童、关心下一代和残疾人事业。

推动文化繁荣发展。把社会主义核心价值体系融入国民教育和精神文明建设全过程，大力弘扬和实践新时期连云港精神，建设和谐文化，实施公民道德建设工程，加强未成年人思想道德建设，广泛开展文明城市、文明村镇、文明行业创建活动，以商务、政务、社会诚信为重点推进诚信连云港建设，提高全社会文明程度。大力发展公益性文化事业，加快完善市博物馆综合功能，推进乡镇文化站和农村文化室建设，有效服务基层文化发展。繁荣文艺创作，推动文化产业发展。加强文化遗产保护，完成全市非物质文化遗产普查。推进市区有线电视数字化。广泛开展全民健身活动，加快县及乡镇体育健身场地设施建设。续修《连云港市志》，市档案馆新馆开工，建设海清寺二期工程。

加强环境保护和生态建设。狠抓污染减排、综合治理和绿化造林。引导化工企业进入园区实施集中管理、集中治污，推动各类开发园区大力开展绿化，积极创建生态工业园、循环经济产业园，省级以上开发区开展环境管理体系认证。突出抓好城镇和工业园区污水处理厂、城市污泥焚烧处理等重点污染减排项目，开展污染源普查，加强农业面源污染防治，实施城市水环境、集中式饮用水源地整治，推进云台山景观生态林建设工程，全市林木覆盖率提高1个百分点。推进土地复垦开发整理，加大矿业秩序整顿力度，开展第二次土地调查。

切实完善社会管理。强化市场监管，加强质量工作，开展食品药品安全整治，保障粮油肉等基本生活必需品和其他紧缺商品的有效供给，努力保持重要消费品和服务价格基本稳定。开展安全生产执法年活动，重点规范部门安全执法和企业安全生产行为，实现安全生产形势持续稳定。完善应急管理机制，提高应急管理水平。完善大信访机制，努力从源头上化解人民内部矛盾。开展法制宣传教育，深入推进和谐社区创建活动，加强社会矛盾纠纷大调解机制、社会治安大防控体系和基层基础建设，推进法治连云港、平安连云港创建，努力维护社会稳定。

六、以服务群众为宗旨，深入推进政府自身建设

本届政府任期已满，新一届政府即将产生。面对新阶段繁重的发展任务，加强政府自身建设是履行好政府职能、促进科学发展的重要保障。必须坚持以人为本、行政为民，坚持科学民主决策、依法行政、加强行政监督，坚持管理创新、制度创新，着力转变职能、提高效能，加快建立行为规范、运转协调、公正透明、廉洁高效的行政管理体制，努力建设服务型政府。

一是坚持为民行政。把实现好、维护好、发展好人民群众的根本利益，作为政府工作的出发点和落脚点，密切与群众的联系，深入了解群众意愿，真诚倾听群众呼声，切实解决群众困难。适应体制转轨、结构转变、社会转型的要求，推进政府机构改革，加快职能转变，强化公共服务和社会管理职能，进一步增加财政对三农、教育、科技、社会保障、医疗卫生等领域的投入，把发展成果更好地体现到提高人民生活水平和质量上，努力满足广大群众改善民生的新期待。

二是深化依法行政。把依法行政贯穿政府决策、执行、监督的全过程和政府管理的各个环节，严格按照法定权限和程序行使权力、履行职责。健全群众参与、专家咨询和集体决策相结合的行政决策和规范性文件制定机制，继续完善城市建设、市场监管、依法行政、新农村建设、社会保障等方面的行政措施。推进政务公开，实施政府新闻发布制度，加快电子政务建设，提高政府工作透明度。强化行政监督，建立行政问责制度，实行严格的责任追究，确保行政规范、政令畅通。

三是推进高效行政。把积极进取的精神和科学务实的态度结合起来，把发展热情和真抓实干结合起来，大兴求真务实之风，大兴调查研究之风，使各项工作做得更加扎实、更富成效，进一步提高政府执行力、公信力。切实精简会议和文件，减少不必要的应酬，把精力用在抓改革、谋发展、保稳定、促和谐上，用在解决群众生产生活的紧迫问题上。改进行政管理方式，深化行政审批制度改革，继续减少行政审批事项，推进部门内部行政审批职能归并，推行网上申报、审批、会办，努力降低行政成本，提高行政效能，改善投资发展软环境。

四是促进清廉行政。认真落实一岗双责，切实加强反腐倡廉建设。坚持用制度管权、按制度办事、靠制度管人，从源头上预防和治理腐败。加强对权力的监督，特别是对领导干部、人财物管理使用、关键岗位的监督。自觉接受市人大法律监督、工作监督和市政协民主监督，认真办理人大代表议案、建议和政协委员提案，加强与各民主党派、工商联、无党派人士和人民团体的联系沟通。进一步加强经济责任审计，规范工程建设招投标、土地和矿产资源出让、政府采购等公共资源管理权力运行。深入推进治理商业贿赂工作，切实解决群众关心的热点难点问题，树立清正廉洁的良好形象。

各位代表，连云港崛起腾飞的任务光荣而艰巨，推动跨越发展的事业催人奋进。让我们紧密团结在以胡锦涛同志为总书记的党中央周围，深入贯彻落实党的十七大精神，在中共连云港市委领导下，解放思想，抓住机遇，开拓奋进，扎实苦干，加快全面奔小康，重振连云港步伐，为建设连云港更加美好的明天而努力奋斗!
